# Umbrella review and meta-analysis of the effect of delayed and immediate pushing in the second stage of labor on neonatal outcomes

**DOI:** 10.1007/s00404-025-08118-z

**Published:** 2025-08-26

**Authors:** Paula Deusa-López, Ferran Cuenca-Martínez, Vanessa Sánchez-Martínez, Núria Sempere-Rubio

**Affiliations:** 1 Department of Physiotherapy, School of Physiotherapy, Universitat de València, Valencia, Spain; 2 Obstetrics Department, Hospital de Dénia, Alicante, Spain; 3 Department of Nursing, School of Nursing and Podiatry, University of Valencia, Valencia, Spain; 4 Frailty and Cognitive Impairment Group (FROG), University of Valencia, Valencia, Spain

**Keywords:** Delayed pushing, Immediate pushing, Second stage of labor, Neonatal outcomes

## Abstract

**Objective:**

To compare neonatal outcomes for immediate pushing and delayed pushing in the second stage of labor in women receiving epidural analgesia.

**Data sources:**

Systematic searches in PubMed, EMBASE, Scopus, and CINAHL without restrictions by language, date of publication, or methodological quality.

**Study selection:**

The inclusion criteria were based on methodological and clinical factors such as population (pregnant women with epidural analgesia), intervention and control (delayed versus immediate pushing), neonatal outcomes, and study design (systematic reviews).

**Data extraction:**

The outcome measures were Apgar scores at 1 and 5 min, neonatal intensive care unit admission, prevalence of low umbilical artery cord pH, and umbilical artery cord pH. The methodological quality was analyzed using the Assessing the Methodological Study Tool for Systematic Reviews (AMSTAR) and Risk Of Bias In Systematic Reviews (ROBIS) scales, and the strength of evidence was established according to the Guidelines Advisory Committee grading criteria. For the umbilical artery cord pH variable, standardized mean differences and 95% confidence intervals were calculated and pooled in a meta-analysis using the random-effects model.

**Data synthesis:**

Seven systematic reviews with meta-analysis were included. The results suggest no difference between groups for Apgar test scores at 5 min, nor in the rate of neonatal intensive care unit admissions. Mixed results were found for delayed pushing leading to improvements in Apgar test scores at 1 min. No statistically significant between-group differences in the umbilical artery cord pH were found. The total duration of the second stage in the delayed pushing group was not significantly correlated with the umbilical artery cord pH.

**Conclusions:**

Delayed pushing produces at least the same neonatal outcomes as immediate pushing in healthy pregnant women receiving epidural analgesia with a single fetus in vertex presentation with a limited quality of evidence.

Review registered in the International Prospective Register of Systematic Reviews PROSPERO (CRD42023397616).

**Supplementary Information:**

The online version contains supplementary material available at 10.1007/s00404-025-08118-z.

## Introduction

### Précis:

Delayed pushing seems to produce the same neonatal outcomes as immediate pushing in healthy women receiving epidural analgesia.

### Callouts:

- There is inconclusive evidence that prolonging the second stage of labor using delayed pushing in women with epidural analgesia affects neonatal outcomes.
- Delayed pushing during the second stage of labor seems to lead to at least the same neonatal outcomes as immediate pushing in women receiving epidural analgesia.
- The meta-analysis revealed no differences in umbilical artery cord pH between the delayed and immediate pushing groups. Therefore, both options carry the same risk of neonatal acidosis.

Friedman defined the second stage of labor in 1955 as the time between full cervical dilation and birth, including a passive phase, with the passive descent of the fetus, and an active phase, which starts when contractions become more intense, or the woman begins to actively push [1]. This definition is widely accepted by authors and expert associations, including the World Health Organization (WHO) [2, 3]. In the second stage of labor, the fetus begins descending with contractions, causing the compression of the rectum and bladder, and prompting a reflex urge to push. The involuntary intrauterine contractions combined with voluntary expulsive efforts activate the respiratory, abdominal, and perineal muscles [4].

The duration of the second stage of labor has long been a topic of debate and considered a factor that could condition birth outcomes [5–7]. Nulliparous women usually complete this stage within three hours, while in subsequent labors it usually concludes within two hours [3]. Besides nulliparity, longer durations are considered appropriate in other situations, including cases involving maternal obesity, high birthweight, induced labor and epidural analgesia [8]. A recent expert review concluded that there is a need for more refined evidence, because the debate over whether neonatal complications are more common when the second stage is longer remains unresolved [7].

The approaches to the second stage of labor influence its duration, and there are two common methods of pushing: immediate pushing and delayed pushing. In immediate pushing, the woman is asked to begin pushing as soon as exploration confirms that the cervix is completely dilated [9]. Delayed pushing involves letting a woman who has completed dilation but does not feel the need to push rest and wait until she does [10]. However, neither is considered the golden standard, and the relationship between maternal pushing and birth outcomes has been the focus of debate for decades [11]. From a physiological perspective, delayed pushing allows a slower and more controlled descent of the fetus and therefore a gradual stretching of the perineal muscles, with less pressure on the anterior vaginal wall, the bladder, the cervical ligaments and connective tissue, since it does not begin until the fetus starts to descend. However, when the expulsive effort begins before the desire to push, this effort creates downward pressure on the vaginal wall, bladder, and the supporting structures in front of the fetal head, which can obstruct fetal descent and contribute to further biomechanical misalignment [12].

Epidural analgesia is widely used and administered to between 10 and 64% of parturient women in high-income countries [13]. An increased duration and medicalization of birth have long been accepted [14], and how the use of epidural analgesia might affect birth outcomes has been the focus of discussion [15, 16]. From the physiological perspective, epidural analgesia blocks sensory and motor neural pathways with a concentration-dependent effect. This seems to reduce the bearing down reflex, thus diminishing the efficacy of pushing efforts, and consequently prolonging the second stage of labor [15, 17]. In women receiving epidural analgesia, the WHO and the NICE recommend delaying pushing for one to two hours after full cervix dilation or until the woman senses the urge to push [18] in association with a second stage that is one hour longer for these women [19–21].

There is a debate regarding the optimal approach to managing the second stage of labor to minimize neonatal complications in women receiving epidural analgesia, and specifically regarding delayed pushing versus immediate pushing. Although the new NICE guideline published in 2023 provides recommendations regarding the type of pushing in women with epidural analgesia, distinguishing between nulliparous and multiparous women, the optimal management of the second stage of labor remains a challenge due to the need to balance maternal and neonatal outcomes with the minimization of unnecessary interventions [22].

Some randomized controlled trials support the hypothesis that delayed pushing might be related to higher one-minute Apgar test scores [9, 23]. However, others suggest an increased risk of neonatal acidosis [24, 25]. Several systematic reviews have compared neonatal outcomes depending on the method of pushing [26–32], but their results are inconclusive. Different neonatal outcomes have been identified as relevant, including Apgar scores, umbilical artery cord pH, and neonatal intensive care unit admission. The lack of consensus regarding the optimal point in time for nulliparous women receiving epidural analgesia to begin pushing highlights the need to compile all the evidence from systematic reviews. The main aim of this umbrella review and meta-analysis was therefore to assess the available evidence regarding different neonatal outcome measures with delayed pushing and immediate pushing in the second stage of labor in women receiving epidural analgesia.

## Methods

This study was reported following the Preferred Reporting Items for Overviews of Systematic Reviews including harms checklist (PRIO-harms), which consists of 27 items (56 sub-items), followed by a five-stage process flow diagram (identification, screening, eligibility, inclusion, and separation of relevant studies) [33] and following PRISMA. This umbrella review was also methodologically guided by the study carried out by Belbasis et al. [5]. The review was previously registered in the International Prospective Register of Systematic Reviews PROSPERO (CRD42023397616]). This review was designed to consider maternal and neonatal outcomes, but the abundance of results led us to divide it into two manuscripts. The maternal outcomes have been published previously [34] ).

### Review question

Do neonatal outcomes differ for immediate and delayed pushing in women with epidural analgesia?

### Review inclusion criteria

The inclusion criteria employed in this article were based on methodological and clinical factors such as population, intervention, control, outcomes and study design (PICOS) [35]. The Population refers to the study subjects. In this case, the population consisted of healthy pregnant women in the second stage of labor who received epidural analgesia. Additionally, as stated below, only women with a single fetus in the vertex presentation were included, regardless of whether they were nulliparous or multiparous. Following the PICOS framework, we identified the Intervention, which was categorized as immediate or delayed pushing. In the former, the woman is guided to start pushing as soon as the cervix is fully dilated, whereas in the latter, the woman pushes when she feels the urge to do so. The Outcomes included clinically relevant neonatal variables, such as Apgar scores (at 1 and 5 min), admission to the neonatal intensive care unit, and pH-related variables. Finally, the methodological designs included in the study were considered. As this is an umbrella review, only systematic reviews with or without a meta-analysis were included. All this information is expanded upon in the following subsections.

### Population

The participants were healthy pregnant women in the active phase of spontaneous labor at term (the second stage of labor) and with a single fetus in the vertex presentation. Only women undergoing epidural analgesia were included, regardless of whether they were nulliparous or multiparous.

#### Intervention and control

The exposures for this review were immediate or delayed pushing in the second stage of labor.

### Outcome measures

For qualitative analysis, each variable must have been evaluated in at least 3 systematic reviews. As a result, the neonatal outcomes included were: 1) Apgar scores at 1 min; 2) Apgar score at 5 min; 3) neonatal intensive care unit admission; 4) low umbilical artery cord pH; 5) umbilical artery cord pH.

### Study design

We selected systematic reviews (with or without a meta-analysis) of randomized controlled clinical trials or controlled clinical trials, and excluded systematic reviews that included non-experimental designs. There were no restrictions for any specific language or by publication date, as recommended by the international criteria [36].

### Search strategy

We searched for published scientific articles (last search on March 31st, 2024), in the following databases: PubMed (Medline), EMBASE, Scopus and CINAHL. The reference sections of the studies included and the original studies were screened manually. The search strategy combined the Medical Subjects Headings: “Labor Stage, Second” [MeSH], or “Delivery, Obstetric” [MeSH Terms], and non-MeSH terms (“Delayed Pushing”, “Passive Descent”, or “Immediate Pushing”, among others), adding a Boolean operator (AND and/or OR) to combine them. Annex 1 shows the search strategies, which were adapted for each database. The reference sections of the original studies were screened manually, and the authors were contacted for further information if necessary.

### Selection criteria and data extraction

Two independent reviewers (PDL and VSM) initially conducted a screening assessing the relevance of the systematic reviews (with and without a meta-analysis) of the studies’ questions and objectives. The first screening was based on each study’s title, abstract, and keywords. The full text was reviewed if there was no consensus or if the abstracts contained insufficient information. In the second phase of the screening, the full text was assessed if the studies met all of the inclusion criteria. The differences between the reviewers were resolved by a discussion and consensus process mediated by a third reviewer (NSR).

### Methodological quality assessment

The two independent reviewers (NSR and FCL) assessed the methodological quality of the systematic reviews (with or without meta-analysis), evaluating each of the studies selected based on the Modified Quality Assessment Scale for Systematic Reviews (AMSTAR) developed by Barton et al. [37], a scale shown to be a valid and reliable tool for assessing the methodological quality of systematic reviews. With a total of 13 items, each worth 2 points (with “yes” scoring 2, “in part” scoring 1; “no” scoring 0), the maximum possible score is 26. The developers provided a high-quality cut-off of 20 or more points. In addition, we calculated the kappa coefficient (*κ*) and percentage (%) agreement scores to assess reliability before any consensus, and estimated the inter-rater reliability using *κ*: 1) *κ* > 0.7 indicates a high level of agreement between the reviewers; 2) *κ* of 0.5–0.7 indicates a moderate level of agreement; and 3) *κ* < 0.5 indicates a low level of agreement [18]. Disagreements on the final quality assessment score were resolved by consensus with a third independent reviewer (PDL).

### Risk of bias assessment

We assessed the risk of bias with the Risk of Bias in Systematic Reviews tool (ROBIS). This consists of 3 phases: 1) a relevance assessment (optional); 2) identification of concerns with the review process through 4 domains related to study eligibility criteria, identification and selection of studies, data collection and study appraisal and synthesis and findings; and 3) judgment on the risk of bias. The ROBIS tool includes signaling questions to evaluate specific domains to help judge the systematic review’s risk of bias, which must be answered as “yes”, “probably yes”, “probably no”, “no” or “no information”. The risk of bias is, therefore, judged as “low”, high” or “unclear” [38]. The two independent reviewers (PDL and VSM) evaluated the risk of bias in the studies selected. Disagreements were resolved through consensus and mediation by a third reviewer (NSR).

### Grading of evidence

The Physical Activity Guidelines Advisory Committee Grading Criteria (PAGAC) were used to assess the grading of evidence. The criteria used to evaluate the quality of the evidence were as follows: 1) applicability of the study sample, exposures, and outcomes to the research question, 2) generalizability to the population of interest, 3) risk of bias/study limitations, 4) quantity and consistency of findings across studies, and 5) magnitude and precision of the effect. The final evidence grades and conclusion statements for each research question were developed based on these data [39].

### Statistical analysis

If the reporting of the umbilical artery cord pH was heterogeneous in the systematic reviews, a specific analysis plan was designed for this variable. Accordingly, a qualitative analysis and an evidence map were used for reviews offering the neonatal acidosis prevalence with consistent criteria, and for reviews offering numeric expressions of umbilical artery cord pH, a meta-analytic approach of the primary studies they included was applied.

The statistical analysis was conducted using the *Meta-Essentials* software package (version 1.5) [40]. When comparing the outcomes reported by the studies, we calculated the standardized mean difference (SMD) over time and the corresponding 95% confidence interval (CI) for the continuous variable umbilical artery cord pH. The statistical significance of the pooled SMD was examined as Hedges’ *g* to account for a possible overestimation of the actual population effect size in the small studies [41].

We used the same inclusion criteria for the systematic review and the meta-analysis, and included 3 additional criteria: 1) the results contained detailed information regarding the comparative statistical data of the exposure factors, therapeutic interventions, and treatment responses; 2) the intervention was compared with a similar comparable group; and 3) data on the analyzed variables were represented in at least 3 studies.

The estimated SMDs were interpreted as described by Hopkins et al. [42], i.e. we considered that an SMD of 4.0 represented an extremely large clinical effect, 2.0–4.0 represented a very large effect, 1.2–2.0 represented a large effect, 0.6–1.2 represented a moderate effect, 0.2–0.6 represented a small effect, and 0.0–0.2 represented a trivial effect [42].

The degree of heterogeneity among the studies was estimated using Cochran’s Q statistical test (a *p*-value <0.05 was considered significant) and the inconsistency index (I^2^) [42]. We considered that an *I*^2^ > 25% represented small heterogeneity, *I*^2^ > 50% represented medium heterogeneity, and *I*^2^ > 75% represented large heterogeneity [43]. The *I*^2^ index complements the Q test, although it has the same power problems with a small number of studies [43]. When the Q-test was significant (*p* < 0.1) and/or the result of *I*^2^ was >75%, there was heterogeneity among the studies, and the random effects model was conducted in the meta-analysis.

A visual evaluation of the funnel plot seeking asymmetry was performed to detect publication biases. If a high risk of publication bias was found, an exclusion sensitivity analysis was performed. Lastly, we applied a meta-regression analysis to analyze the relationship between the total duration of the second stage (minutes) in the delayed pushing group and umbilical artery cord pH using a random effects model, employing the effect size statistic (Hedges’ *g*) of the outcome score to correlate with the total duration of the second stage (minutes) in the delayed pushing group [40].

### Evidence map

To visually display the information, we created a visual map of the scientific evidence for each systematic review (with and without meta-analysis). The review information is based on four dimensions [23].

1. Number of studies (triangle size): the size of each triangle is directly proportional to the number of original studies included in each of the systematic reviews.
  Outcome measures (triangle color): each assessed variable was determined by color: 1) Apgar scores at 1 minute (blue); 2) Apgar score at 5 minutes (green); 3) Neonatal intensive care unit admission (gold).
2. Number of studies (bubble size): the size of each bubble is directly proportional to the number of original studies included in our quantitative results. Outcome measure: umbilical artery cord pH (pink).
3. Effect size (x-axis): the authors classified each review according to the effects found. When the delayed pushing showed greater benefits than the immediate pushing, the intervention was classified as “potentially better”; otherwise, the intervention was classified as “potentially worse”. When there was insufficient evidence, the intervention was classified as “unclear”. If there were no differences, the intervention was classified as “no differences”. If there were contradictory results, we classified the intervention as “mixed results”.
4. Strength of findings (y-axis): AMSTAR.

## Results

### Study selection

**Fig. 1:**
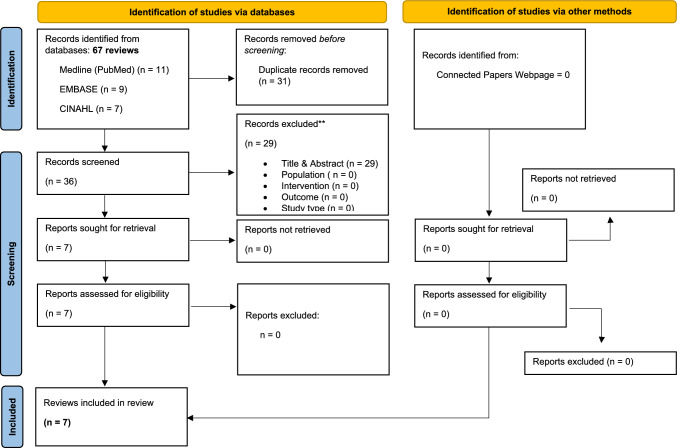
PRISMA Flowchart of studies selection

The initial search revealed 67 records; no additional study was retrieved manually from the references. Through the title, abstract screening, and full-text assessment, seven systematic reviews were eligible according to our criteria. The study screening strategy is shown as a PRISMA diagram (Fig. 1).

### Characteristics of the systematic reviews included

Table 1 lists the characteristics of the systematic reviews’ (study design, original studies included, demographic characteristics, interventions, variables, and results). Considering time, the operational definitions of delayed pushing included 1, 2 or 3 h, and from 1 to 3 h, or the presence of the involuntary pressure/urge to push in this timeframe.

**Table 1:** Characteristics of the reviews included

Reviews	Number of RCTs (total sample)	Definition of DP	Outcomes measures	Results
Szu et al. [31]	15 RCTs (6121)	DP from 1 to 3 h	– Apgar scores (at 1 min) – Apgar scores (at 5 min)	In the DP group the Apgar scores at 1 min were higher to the IP group. However, Apgar scores at 5 min did not differ between the IP and DP groups
Di Mascio et al. [27]	12 RCTs (5445)	DP from 1 to 3 h	– Umbilical artery cord pH – Apgar scores (at 5 min) – Neonatal intensive care unit admission	In the DP group they found a significantly higher incidence of low umbilical artery cord pH, but no difference in Apgar score <7 at 5 min or neonatal intensive care unit admission
Lemos et al. [28]	20 RCTs (3694)	DP was until the woman experienced an irresistible urge to push, or to 1, 2 or 3 h	– Neonatal intensive care unit admission – Apgar scores (at 5 min) – Umbilical artery cord pH	There were no clear differences between IP or DP groups in Neonatal intensive care unit admission and low Apgar score at 5 min. In the DP group, the risk of low umbilical artery cord blood pH was higher than IP
Tuuli et al. [32]	12 RCTs (3115)	DP from 1 to 3 h or in the presence of involuntary pressure/urge to bear down	– Umbilical artery cord pH	There were no adverse results on low Apgar and low umbilical artery cord blood pH in the DP group
Brancato et al. [26]	7 RCTs (2827)	DP up to 2 h	– Apgar scores – Neonatal intensive care unit admission – Umbilical artery cord pH	It was not possible to draw definitive conclusions
Menez-Orieux et al. [29]	8 RCTs (4732)	DP from 1 to 3 h	– Umbilical artery cord pH – Apgar scores (at 1 min) – Apgar scores (at 5 min)	It was not possible to draw definitive conclusions
Roberts et al. [30]	9 RCTs (3000)	DP from 1 to 3 h or in the presence of involuntary pressure/urge to bear down	– Apgar scores (at 1 min) – Apgar scores (at 5 min) – Umbilical artery cord pH – Neonatal intensive care unit admission	There were no adverse results on low Apgar and low umbilical artery cord blood pH in the DP group

*RCTs* Randomized Controlled Trials, *DP* Delayed Pushing, *IP* Immediate Pushing

### Methodological quality results (AMSTAR)

The scores ranged from 13 to 26 points out of a possible 26, with a mean score of 18.7 ± 4.4 points. Only 3 (43%) studies scored above 20 points and were considered high-quality (Table 2). The items with the highest scores were those related to “explicitly described to allow replication”, and “meta-analysis conducted on only homogeneous data or limitations to homogeneity discussed”. The lowest scoring item was “conclusions address levels of evidence for each intervention/comparison”. The inter-rater reliability of the methodological quality assessment was high (*κ* = 0.816). The study with the highest score was the one conducted by Lemos et al. [28], while the study with the lowest score was the one undertaken by Menez-Orieux et al. [29].

**Table 2:** Quality assessment scores (AMSTAR)

Study	1	2	3	4	5	6	7	8	9	10	11	12	13	Score
Szu et al. [31]	2	1	1	2	1	2	2	2	2	2	2	1	0	**21**
Di Mascio et al. [27]	2	2	1	1	2	2	2	2	2	2	2	2	2	**24**
Lemos et al. [28]	2	2	2	2	2	2	2	2	2	2	2	2	2	**26**
Tuuli et al. [32]	2	0	0	2	2	1	1	2	1	2	2	1	1	**17**
Brancato et al. [26]	1	1	2	2	0	1	0	0	0	2	2	2	1	**14**
Menez-Orieux et al. [29]	2	1	2	0	1	1	1	2	0	2	0	1	0	**13**
Roberts et al. [30]	2	2	1	1	2	1	1	2	0	1	2	1	0	**16**

Items: 1. Explicitly described to allow replication; 2. Adequate number and range of databases; 3. Alternative searches; 4. Adequate range of keywords; 5. Non-English-language papers included in the search; 6. Inclusion criteria explicitly described to allow replication; 7. Excludes reviews which do not adequately address inclusion and exclusion criteria; 8. Two independent reviewers assessing selection bias; 9. Quality assessment is explicitly described to allow replication; 10. Meta-analysis conducted on only homogeneous data or limitations to homogeneity discussed; 11. Confidence intervals/effect sizes reported where possible; 12. Conclusions supported by the meta-analysis or other data analysis findings; 13. Conclusions address levels of evidence for each intervention/comparison

### Risk of bias

**Fig. 2:**
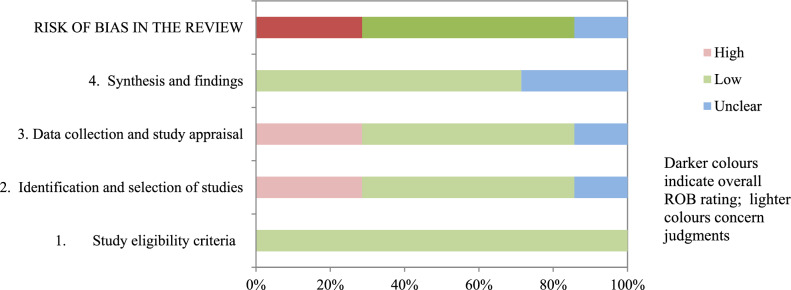
Graphical representation for ROBIS results

Table 3 and Fig. 2 show the results of the risk of bias assessment using ROBIS. 57.1% of the studies had a low risk of bias. The domains related to the “study eligibility criteria” and the “synthesis and findings” had the lowest risk of bias. In contrast, the domain related to the “identification and selection of studies”, and “data collection and study appraisal” had the highest risk of bias. The inter-rater reliability for the risk of bias assessment was high (*κ* = 0.891).

**Table 3:** Risk of bias assessment in systematic reviews through ROBIS scale

Study	Phase 2				Phase 3
	1. Study eligibility criteria	2. Identification and selection of studies	3. Data collection and study appraisal	4. Synthesis and findings	Risk of bias in the review
Szu et al. [31]					Low
Di Mascio et al. [27]					Low
Lemos et al. [28]					Low
Tuuli et al. [32]					Unclear
Brancato et al. [26]					High
Menez-Orieux et al. [29]					High
Roberts et al. [30]					Low

: low risk. : high risk. : unclear risk

### Grading of evidence results (PAGAC)

**Fig. 3:**
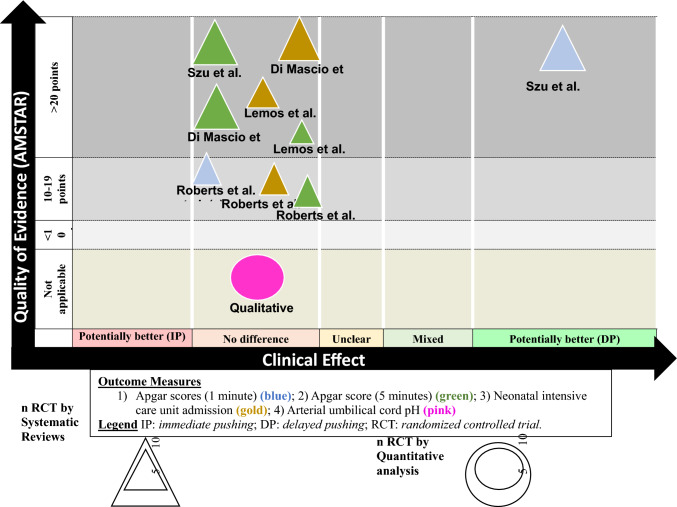
Evidence map

Table 4 shows the findings regarding the quality of evidence for each outcome of the research question. The quality of evidence found for all outcome measures was limited. The evidence map is represented in Fig. 3.

**Table 4:** Summary of findings and quality of evidence (PAGAC)

Guidelines Advisory Committee Grading Criteria						Grade
Systematic Review Research Questions	Applicability	Generalizability	Risk of bias or study limitations	Quantity and consistency	Magnitude and precision of effect	
Apgar scores (1′ and 5′)	Strong	Limited	Limited	Limited	Not applicable	Limited
Umbilical cord pH	Strong	Limited	Limited	Limited	0.24 (−0.22 to 0.71)	Limited
Neonatal intensive care unit admission	Strong	Limited	Limited	Limited	Not applicable	Limited

### Qualitative results

#### Apgar scores at 1 min

A total of 2 systematic reviews assessed the Apgar scores at 1 min [30, 31]. Szu et al. [31] found that the delayed pushing group scores were significantly higher, by 0.19 points, compared to the immediate pushing group (*n* = 6, mean differences (MD = 0.19, 95% CI 0.10–0.27, *I*^2^ = 0%). However, [30] did not show any significant differences between the immediate pushing and delayed pushing groups (*n* = 3, risk ratio (RR) = 0.96, 95% CI 0.74–1.24).

#### Apgar scores at 5 min

A total of 5 systematic reviews assessed the Apgar scores at 5 min [27–31]. Except for Menez-Orieux et al. [29], which cannot draw definitive conclusions for the Apgar scores at 5 min, all the systematic reviews found no significant differences between the immediate pushing and delayed pushing groups [31]: *n* = 6, MD = 0.08, 95% CI −0.05–0.20, *I*^2^ = 0%, [27]: n = 6, MD = 0.15, 95% CI 0.01–3.00, *I*^2^ = 0%, [30]: *n* = 3, RR = 0.82, 95% CI 0.50–1.36, [28]: n = 1, RR = 0.15, 95% CI 0.01–3.00, *I*^2^ = not applicable).

#### Neonatal intensive care unit admission

A total of 3 systematic reviews assessed neonatal intensive care unit admission [27, 28, 30]. All the systematic reviews found no significant differences between the immediate pushing and delayed pushing groups related to the need for admission to neonatal intensive care [27]: *n* = 6, RR = 1.12, 95% CI 0.08–1.42, *I*^2^ = 0%, [30]: *n* = 4, RR = 1.00, 95% CI 0.70–1.42, and [28]: *n* = 3, RR = 0.98, 95% CI 0.67–1.41, *I*^2^ = 0%).

#### Prevalence of low umbilical artery cord pH

Four systematic reviews assessed the presence of low umbilical artery cord pH [26–28, 32]. Although two of these systematic reviews considered a threshold of 7.2 among their outcome measure description [27, 28], only one of the primary studies these reviews included contained this condition [44], and they included other studies for which low pH was under 7.1. More precisely, the two largest RCTs they included considered a threshold of 7.1 [24, 25]. The other two systematic reviews operationalized low pH when the rates were below 7.1 [26, 32]. In view of these disparities between the selection criteria described by the authors and the inclusion of studies that did not meet them, the qualitative analysis and the evidence map were not undertaken for this outcome.

### Quantitative results

#### Umbilical artery cord pH

Three systematic reviews [28, 30, 32] considered pH as a numeric expression among the outcome measures included in their systematic reviews, but only Roberts et al. [30] developed a meta-analysis for this outcome measure. For this reason, a new meta-analysis was created, including the numeric results of pH reported in the primary studies included in the systematic reviews considered in this umbrella review.

**Fig. 4:**
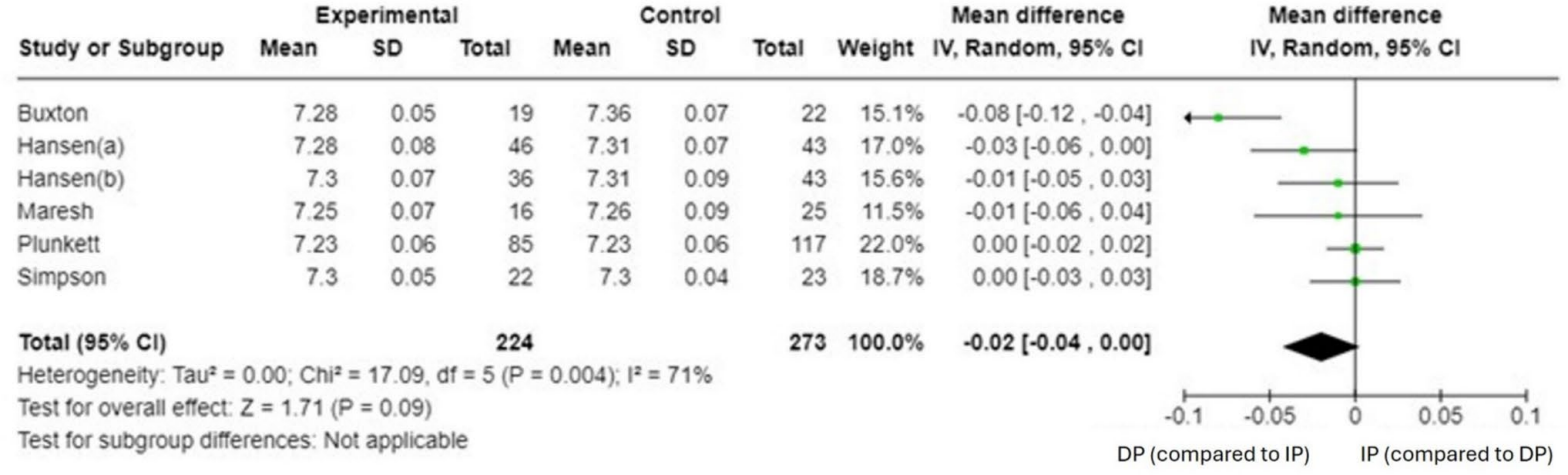
Synthesis forest plot of umbilical artery cord pH. Notes: Positive values (right) indicate a higher umbilical artery cord pH for immediate pushing (compared to delayed pushing). Negative values (left) indicate a lower umbilical artery cord pH for delayed pushing (compared to immediate pushing). As the 95% confidence interval passes through 0, the results are not statistically significant between groups

**Fig. 5:**
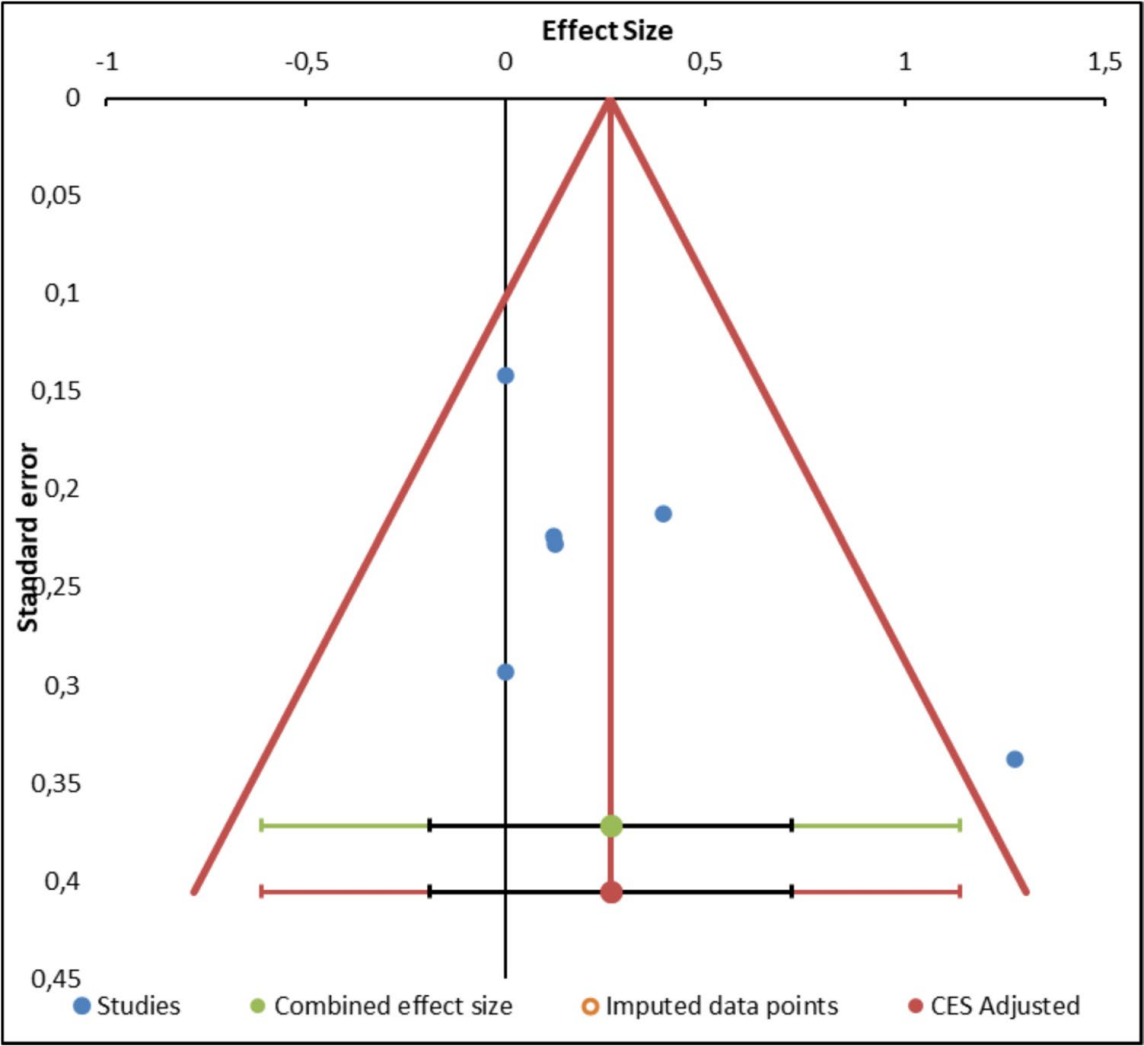
Synthesis Funnel Plot for umbilical artery cord pH. The funnel plot shows marked asymmetry, indicating potential publication bias. The distribution of the effect sizes is uneven, with a clustering of smaller studies showing larger effects

**Fig. 6:**
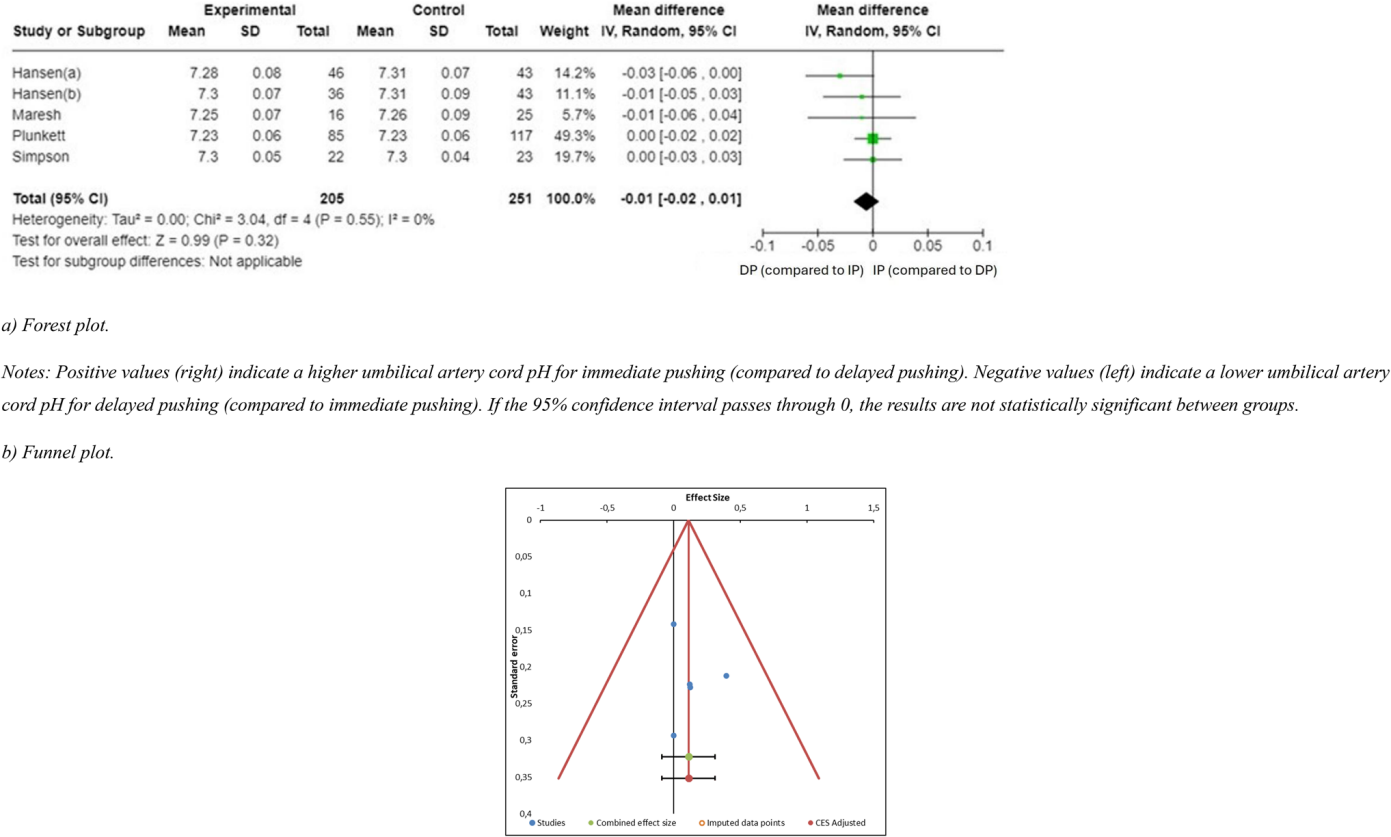
Sensitivity Exclusion Analysis for umbilical artery cord pH (**a**: forest plot, **b** funnel plot). **a** Forest plot. Notes: Positive values (right) indicate a higher umbilical artery cord pH for immediate pushing (compared to delayed pushing). Negative values (left) indicate a lower umbilical artery cord pH for delayed pushing (compared to immediate pushing). If the 95% confidence interval passes through 0, the results are not statistically significant between groups. **b** Funnel plot

The primary studies offering numeric expressions of pH [9, 44–47] were considered when performing this meta-analysis (Annex 2). The meta-analysis found no statistically significant between-group differences in umbilical artery cord pH (*n* = 6 (497 women), SMD = −0.02 (95% CI −0.04 to 0.00), *p* = 0.09, *I*^2^ = 71% (Fig. 4). In addition, the funnel plot showed marked asymmetry, indicating potential publication bias. The distribution of the effect sizes was uneven, with a clustering of smaller studies showing larger effects. This was supported by the presence of significant heterogeneity (*Q* = 13.62, *p* = 0.018), suggesting that the variability between studies was not solely due to chance (Fig. 5). Because of this high risk of publication bias, a sensitivity analysis was carried out to exclude the article conducted by Buxton et al. [44]. The sensitivity analysis revealed no evidence of significant between-group differences in the umbilical artery cord pH (*n* = 5 (456 women), SMD = −0.01 (95% CI −0.02 to 0.01), *p* = 0.32, *I*^2^ = 0%, with no evidence of significant heterogeneity (*Q* = 2.55, *p* = 0.63) (Fig. 6. A meta-regression analysis was performed to investigate the association between the total duration of the second stage of labor (in minutes in the delayed pushing group and umbilical artery cord pH. Although the regression coefficient indicated a positive trend (*β* = 0.66, this relationship did not reach statistical significance (*Z* = 1.9; *p* = 0.053. The model accounted for 43.84% of the between-study variance (*R*^2^ = 43.84%), suggesting moderate explanatory power. These findings imply that, based on the available data, the duration of the second stage in delayed pushing is not significantly associated with changes in umbilical artery cord pH (Annex 3).

## Discussion

This umbrella review and meta-analysis aimed to assess the evidence obtained from systematic reviews of neonatal outcome measures comparing immediate pushing and delayed pushing in the second stage of labor in women receiving epidural analgesia. With a limited amount of evidence, our findings indicate that there are no differences in neonatal outcomes that favor immediate pushing compared to delayed pushing. More precisely, the mixed results suggested that delayed pushing might favor the Apgar score at 1 min, and we found no differences in the Apgar test scores at 5 min, the rates of NICU admissions, or the umbilical artery cord pH. The meta-regression results also suggested that the duration of the second stage did not influence umbilical artery cord blood pH.

Regarding the umbilical artery cord pH, our findings are in line with the results obtained by Roberts et al. [30] and in contrast with Di Mascio et al. [27] and Lemos et al. [28]. The decision to undertake a new meta-analysis to study the arterial cord pH was made after finding inconsistencies when the results from the systematic reviews were combined, given the lack of an accepted threshold for low umbilical artery cord pH. Roberts et al. [30] used the weighed mean difference to calculate the effect size, using the mean values obtained by groups in the primary studies they considered [9, 44, 46]. In contrast, Di Mascio et al. [27] and Lemos et al. [28] only considered studies reporting the prevalence of low cord blood pH [24, 25, 44, 46, 48] with the limitation that these studies categorized pH with different cut-off points to consider the presence of acidemia. For this reason, they could only calculate risk ratios but not determine if the pH differed between the immediate and delayed pushing groups.

The discrepancy regarding an accepted threshold for umbilical artery blood pH has previously been discussed. Olofsson [49] concluded in his review that there is a lack of consensus on definitions of standard cord blood gases and that a variety of thresholds for abnormality has been used, but establishing a general critical pH cut-off is challenging. Malin et al. [50] produced a systematic review including 479,022 infants, and the 46 primary studies they combined used heterogeneous thresholds for arterial cord pH, mostly ranging from 7.00 to 7.24, thereby limiting their opportunities to explore the effect of varying pH levels.

Other than the existence of accepted thresholds, some studies even question the relevance of cord blood pH values. Examples include Heller et al. [51], who produced a large retrospective cohort study including 513,135 live births in 2003, and concluded that Apgar scores at 1, 5, and 10 min were better predictors for early neonatal mortality than any umbilical blood pH cut-off used. After their cohort study, Yeh et al. [52] also reported that low pH was not a good predictor of neonatal neurological outcomes, as more than 75% of neonates with neurological outcomes examined had pH values above 7.10. Likewise, Gonen et al. [53] concluded in their review that in low-risk births, umbilical artery cord blood studies were uncommon and questioned the clinical relevance of their association with composite adverse neonatal outcomes.

A reflection on the use of the duration of the second stage of labor as the sole criterion for intervening in labor might be reductionist. The decision to intervene should not only be based on time [6, 54], but also on a balanced assessment of the fetal status, the risk associated with the mode of birth, the presence of maternal complications and taking maternal preferences into account, with a view to providing a more humanized care in labor. This is even more relevant in contexts in which labor is highly medicalized [55], such as high-income countries, which have experienced a substantial increase in the active management of childbirth in terms of initiating, accelerating, terminating, regulating, or monitoring the physiological process to improve labor outcomes for women and neonates. Although medicalization has been helpful for some outcomes, interfering with the normal process of labor without medical justification should be questioned, as it may also have adverse effects on the woman and neonate [56]. Epidural analgesia involves administering local anesthetics into the epidural space, producing dose-dependent sensory and motor effects. While higher concentrations can cause complete motor block and limit mobility—potentially reducing the bearing down reflex and prolonging labor stages [10]—current practice favors lower doses combined with opioids. This approach enhances analgesia while preserving some motor function, allowing women to remain mobile and maintain their ability to push during the second stage of labor [57].

### Limitations

This umbrella review reached conclusions based on a limited quality of evidence, related to the systematic reviews included and the primary studies they comprised. This limited quality of the evidence available means that rigorous new studies, including large samples may provide different results. If the quality of the primary studies included in the systematic reviews was higher, the findings of the systematic reviews would be unlikely to be greatly modified by subsequent studies.

Four limitations of this umbrella review were: 1) five randomized controlled trials involving 2,629 women had been included in all the systematic reviews; 2) the diversity of the timeframes used to define immediate and delayed pushing, ranging from 1 to 3 h; 3) the heterogeneity of techniques to administer epidural analgesia; and 4) the inclusion of women with and without induced labor and at different gestational ages should also be considered [10]. In addition, this umbrella review was limited by the inability to analyze the differential effects of parity (nulliparous vs. multiparous women) on the duration of the second stage of labor and related outcomes. This was because the included systematic reviews generally did not report stratified results by parity, which restricted our capacity to explore this potentially important clinical variable.

The results of this umbrella review could be generalizable to women receiving epidural analgesia during childbirth, but not to those with physiological and non-interventional childbirth.

### Implications for practice and research

According to our findings, the duration of the second stage of labor does not seem to negatively influence neonatal outcomes in healthy pregnant women receiving epidural analgesia with a single fetus in the vertex presentation. However, the limited quality of the available evidence obliges us to be cautious in this affirmation.

Prolonging the second stage of labor is generally safe, bearing in mind that the beginning of pushing should not be postponed for more than 2 h, because delayed pushing does not seem to offer benefits for the mother after that time [34].

Studies reporting information that can be categorized or numeric values could increase their transparency by providing both options. For example, low umbilical artery cord blood pH data was reported as a categorized variable (low or otherwise) in the two largest RCTs included in the systematic reviews [24, 25]. This is even more relevant when experts discuss the thresholds assumed. The fact that these studies reported only categorized data prevented us from considering their valuable results in the meta-analysis undertaken as a part of this review.

Future studies considering the duration of the second stage of labor should also consider the description of other relevant variables in the duration of the second stage of labor. Some of these relevant variables, like the description of the fetal station and position at the beginning of the second stage, are frequently omitted in the studies; an initially high fetal station or a fetal malposition might have confounded the primary outcome. The heterogeneity of techniques for administering epidural analgesia and the inclusion of women undergoing induced labor or otherwise, and at different gestational ages should also be considered [10]. There is also a need for longitudinal studies in which the duration of the second phase of labor is related to long-term infant outcomes, such as neurodevelopmental parameters. Finally, this study explicitly provides a comprehensive integration of all available publications to date analyzing the relationship between the duration of the second stage of labor and neonatal outcomes in healthy pregnant women receiving epidural analgesia. Moreover, it critically addresses an important methodological issue related to the assessment of umbilical cord blood pH, highlighting how presenting this variable as either categorical or continuous can significantly influence the results and their interpretation. Thanks to this methodological rigor and the inclusion of updated data, our work offers solid and precise clinical evidence that helps clarify a question that has remained unresolved for decades, thereby guiding future research and clinical practice.

## Conclusions

The present umbrella review found no significant differences between immediate and delayed pushing in the second stage of labor regarding 5-min Apgar scores or neonatal intensive care admissions, with limited evidence quality. Mixed results were observed for 1-min Apgar scores in favor of delayed pushing, though evidence remains limited. No definitive conclusion could be drawn about low umbilical artery cord pH due to inconsistent cut-off criteria. Additionally, delayed pushing was not associated with increased umbilical artery acidification, and longer durations did not correlate with greater acidification. Overall, neonatal outcomes appear neither improved nor worsened by immediate or delayed pushing, and current evidence does not support immediate pushing to enhance these outcomes.

## Supplementary Information

Below is the link to the electronic supplementary material.

Supplementary file 1 (DOCX 35 KB)

## Data Availability

No datasets were generated or analysed during the current study.
