# Supplementary material for: Umbrella review and meta-analysis of the effect of delayed and immediate pushing in the second stage of labor on neonatal outcomes
Source: Arch Gynecol Obstet. 2025 Aug 26;312(5):1419–33. doi: 10.1007/s00404-025-08118-z (PMC12589254; doi:10.1007/s00404-025-08118-z)
Supplement: Supplementary file 1 — (DOCX 35 KB) [file 404_2025_8118_MOESM1_ESM.docx]

**Annex 1.** Database search strategies

**P:** Women in the second stage of labour

**I:** Delayed pushing

**C:** Immediate pushing

**O:** Apgar score at 1 minute, Apgar score at 5 minutes, admissions to neonatal intensive care units (NICU), and umbilical artery cord pH.

**S:** Systematic review with and without meta-analysis

**Medline (PubMed)**

("delivery, obstetric"[MeSH Terms] OR "labor stage, second"[MeSH Terms] OR ("labor stage, second"[MeSH Terms] OR ("labor"[All Fields] AND "stage"[All Fields] AND "second"[All Fields]) OR "second labor stage"[All Fields] OR "labor stage second"[All Fields]) OR ("labor stage, second"[MeSH Terms] OR ("labor"[All Fields] AND "stage"[All Fields] AND "second"[All Fields]) OR "second labor stage"[All Fields] OR ("labor"[All Fields] AND "stages"[All Fields] AND "second"[All Fields])) OR ("second labour stage"[All Fields] OR "labor stage, second"[MeSH Terms] OR ("labor"[All Fields] AND "stage"[All Fields] AND "second"[All Fields]) OR "second labor stage"[All Fields] OR ("second"[All Fields] AND "labor"[All Fields] AND "stage"[All Fields])) OR ("second labour stages"[All Fields] OR "labor stage, second"[MeSH Terms] OR ("labor"[All Fields] AND "stage"[All Fields] AND "second"[All Fields]) OR "second labor stage"[All Fields] OR ("second"[All Fields] AND "labor"[All Fields] AND "stages"[All Fields]) OR "second labor stages"[All Fields]) OR ("labor stage, second"[MeSH Terms] OR ("labor"[All Fields] AND "stage"[All Fields] AND "second"[All Fields]) OR "second labor stage"[All Fields] OR ("stage"[All Fields] AND "second"[All Fields] AND "labor"[All Fields])) OR ("labor stage, second"[MeSH Terms] OR ("labor"[All Fields] AND "stage"[All Fields] AND "second"[All Fields]) OR "second labor stage"[All Fields] OR ("stages"[All Fields] AND "second"[All Fields] AND "labor"[All Fields])) OR ("labor stage, second"[MeSH Terms] OR ("labor"[All Fields] AND "stage"[All Fields] AND "second"[All Fields]) OR "second labor stage"[All Fields] OR ("labor"[All Fields] AND "second"[All Fields] AND "stage"[All Fields]) OR "labor second stage"[All Fields]) OR ("second stage labour"[All Fields] OR "labor stage, second"[MeSH Terms] OR ("labor"[All Fields] AND "stage"[All Fields] AND "second"[All Fields]) OR "second labor stage"[All Fields] OR ("second"[All Fields] AND "stage"[All Fields] AND "labor"[All Fields]) OR "second stage labor"[All Fields]) OR "Second Stage of Labor"[Title/Abstract] OR "Second Stage of Delivery"[Title/Abstract]) AND ((("delay"[All Fields] OR "Delayed"[All Fields] OR "delaying"[All Fields] OR "delays"[All Fields]) AND ("pushed"[All Fields] OR "pushes"[All Fields] OR "pushing"[All Fields])) OR "delayed pushing"[Title/Abstract] OR (("Immediate"[All Fields] OR "immediately"[All Fields]) AND ("pushed"[All Fields] OR "pushes"[All Fields] OR "pushing"[All Fields])) OR "immediate pushing"[Title/Abstract] OR (("Spontaneous"[All Fields] OR "spontaneously"[All Fields]) AND ("pushed"[All Fields] OR "pushes"[All Fields] OR "pushing"[All Fields])) OR "spontaneous pushing"[Title/Abstract] OR (("direct"[All Fields] OR "Directed"[All Fields] OR "directing"[All Fields] OR "direction"[All Fields] OR "directional"[All Fields] OR "directions"[All Fields] OR "directivities"[All Fields] OR "directivity"[All Fields] OR "directs"[All Fields]) AND ("pushed"[All Fields] OR "pushes"[All Fields] OR "pushing"[All Fields])) OR "directed pushing"[Title/Abstract]) AND ("systematic review"[Publication Type] OR "systematic review"[Title/Abstract] OR "Meta-Analysis"[Publication Type] OR "Meta-Analysis"[Title/Abstract])

**EMBASE**

('labor stage 2'/exp OR 'delivery stage ii' OR 'labor stage 2' OR 'labor stage ii' OR 'labor stage, second' OR 'labour stage 2' OR 'labour stage ii' OR 'labour stage, second' OR 'second delivery stage' OR 'second labor stage' OR 'second labour stage' OR 'second stage of delivery' OR 'second stage of labor' OR 'second stage of labour' OR 'women with epidural analgesia') AND ('delayed pushing'/exp OR 'spontaneous pushing' OR 'delayed bearing down') AND ('immediate pushing' OR 'directed pushing' OR 'immediate bearing down') AND ('systematic review'/exp OR 'review, systematic' OR 'systematic review' OR 'meta analysis'/exp OR 'analysis, meta' OR 'meta analysis' OR 'meta-analysis' OR 'metaanalysis')

**CINAHL Complete (through the *University of Valencia*)**

(second stage of labor or second stage of labor or vaginal birth or vaginal delivery) AND ( delayed pushing and immediate pushing ) OR ( directed pushing or spontaneous pushing ) OR bearing-down efforts OR passive descent AND ( systematic reviews or meta analysis or meta-analysis )

**Scopus**

(TITLE-ABS-KEY("second stage of labor" OR "labor, second stage" OR "second phase of labor" OR "second stage of delivery" OR "second phase of delivery")) AND (TITLE-ABS-KEY-AUTH("Delayed pushing" OR "Immediate pushing" OR "Spontaneous pushing" OR "Directed pushing" OR "Passive descent")) AND (TITLE-ABS-KEY("Systematic review" OR "Meta-analysis"))

**Annex 2.** Umbilical artery pH data.

| Study | DP: Mean ± SD (n) | IP: Mean ± SD (n) | Duration second stage of labor (minutes) |
| --- | --- | --- | --- |
| Buxton, 1988 | 7.28±0.05 (19) | 7.36±0.07 (22) | 209 |
| Plunkett, 2003 | 7.23±0.06 (85) | 7.23±0.07 (117) | 99 |
| Hansen, 2002a | 7.28±0.08 (46) | 7.31±0.07 (43) | 171 |
| Hansen, 2002b | 7.30±0.07 (36) | 7.31±0.09 (43) | 62.9 |
| Maresh, 1983 | 7.25±0.07 (16) | 7.26±0.09 (25) | 170 |
| Simpson, 2005 | 7.30±0.05 (22) | 7.30±0.04 (23) | 139 |
| Fraser, 2000 | *N/R* | *N/R* | 187 |
| Vause, 1998 | *N/R* | *N/R* | *N/R* |
| Cahill, 2018 | *N/R* | *N/R* | 134.2 |
| Mayberry, 1999 | *N/R* | *N/R* | 119.6 |

*Abbreviations. DP: Delayed pushing; IP: Immediate pushing; n: sample size; N/R: Not reported.*

***SYSTEMATIC REVIEWS***

**1-Lemos et al.**

**-Criteria:** Arterial blood pH<7.2 and venous less than 7.3

**-Results:** The risk of a low umbilical cord blood pH was higher with the use of delayed compared to immediate pushing (RR 2.24, 95% CI 1.37 to 3.68; four studies; 2145 women; I² = 0%).

**2-Di Mascio et al.**

**-Criteria:** “As defined by authors” a) arterial umbilical cord pH <7.2 or b) less as a threshold to define low pH.

**-Result:** A significantly higher incidence of low umbilical cord Ph was found in the delayed pushing group (2.7%vs 1.3%; RR, 2.00; 95%CI, 1.30 3.07; 5RCTs, 4549 women).

**3-Roberts et al.**

**-Criteria:** Not Reported.

**-Result:** No statistically significant between difference were found (WMD: 0.03 (-0.01 to 0.06); 411 women.

**4. Tuuli et al.**

**-Criteria:** Abnormal cord pH was defined as venous pH less than 7.15 or arterial pH less than 7.10

**-Result:** Umbilical cord pH and gases were also variously measured and reported. Although some studies reported no differences in abnormal cord pH or gases, the largest study reported a significantly higher rate of abnormal cord pH (4.5% compared with 1.8%, RR 2.45, 1.35– 4.43) in the delayed pushing group. Two other studies also reported a higher rate of abnormal cord gases in the delayed pushing group, although not statistically significant.

**5. Brancato et al.**

**-Criteria:** Abnormal cord pH was defined as venous pH less than 7.15 or arterial pH less than 7.10.

**-Result:** With the exception of Fraser et al., there were no differences in umbilical cord pH.

**Links to primary studies**

**-Buxton, 1988:** <https://www.tandfonline.com/doi/epdf/10.3109/01443618809012295?needAccess=true>

**-Plunkett, 2003:** <https://pubmed.ncbi.nlm.nih.gov/12850615/>

**-Hansen, 2002a/b:** <https://pubmed.ncbi.nlm.nih.gov/11777506/>

**-Maresh, 1983:** <https://pubmed.ncbi.nlm.nih.gov/6871129/>

**-Simpson, 2005:** <https://pubmed.ncbi.nlm.nih.gov/15897790/>

**-Cahill, 2018:** <https://jamanetwork.com/journals/jama/fullarticle/2706136>

**-Vause, 1998:** <https://pubmed.ncbi.nlm.nih.gov/9501784/>

**-Fraser, 2000:** <https://pubmed.ncbi.nlm.nih.gov/10819854/>

**Annex 3.** Meta-regression analysis to analyse the relationship between the total duration of the second stage (minutes) in the delayed pushing group and umbilical artery cord pH.


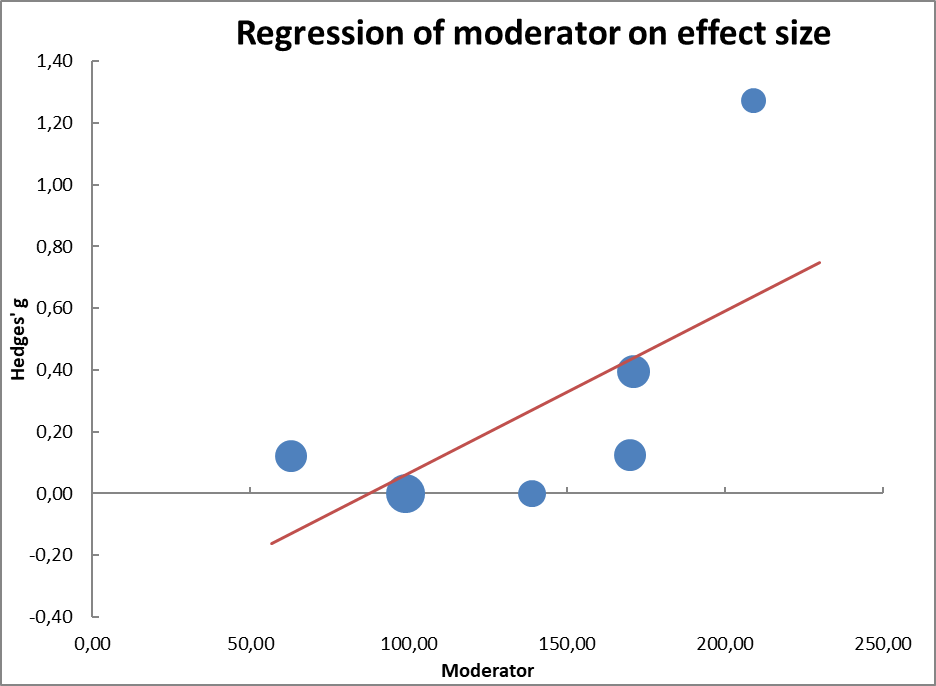


These findings imply that, based on the available data, the duration of the second stage in delayed pushing is not significantly associated with changes in umbilical artery cord pH.
